# Mechanism prediction of Wenjing Tongluo Powder for post-laparoscopic shoulder pain: A network pharmacology study

**DOI:** 10.1097/MD.0000000000048399

**Published:** 2026-04-24

**Authors:** Zhaoyang Wei, Hui Yu, Shen’ao Yu, Yanru Wang

**Affiliations:** a School of Nursing, Zhejiang University of Traditional Chinese Medicine, Hangzhou, Zhejiang Province, China; b Jinhua Hospital of Traditional Chinese Medicine, Jinhua, Zhejiang Province, China.

**Keywords:** network pharmacology, PI3K/AKT signaling pathway, postoperative scapular pain after laparoscopy, Wenjing Tongluo Powder

## Abstract

This study aimed to predict the pharmacological mechanisms of Wenjing Tongluo Powder (WTP) in relieving post-laparoscopic shoulder pain (PLSP) using network pharmacology, and to provide prioritized candidate targets and pathways for future experimental validation. Active ingredients of WTP were screened from the Traditional Chinese Medicine Systems Pharmacology Database and Analysis Platform database using ADME criteria (oral bioavailability ≥ 30%, drug-likeness ≥ 0.18). Their potential targets were predicted via the SwissTargetPrediction database. PLSP-related targets were retrieved from disease databases (Online Mendelian Inheritance in Man, therapeutic target database, etc). The intersecting targets were analyzed through protein–protein interaction network construction (using STRING and Cytoscape) to identify hub genes, followed by Gene Ontology and Kyoto Encyclopedia of Genes and Genomes pathway enrichment analyses. A total of 72 active ingredients and 546 potential targets of WTP were identified. 136 common targets intersected with PLSP-related targets. Protein–protein interaction network analysis highlighted COX-2, TNF-α, ESR1, EGFR, CASP3, BCL2, MAOA, MAOB, and PPARG as core targets. Gene Ontology analysis suggested involvement in processes like vascular regulation and G protein-coupled receptor activity. Kyoto Encyclopedia of Genes and Genomes enrichment analysis predicted that key pathways, including the calcium signaling pathway, cAMP signaling pathway, PI3K-Akt signaling pathway, and cGMP-PKG signaling pathway, may be crucial for the therapeutic effect. This network pharmacology study predicts that WTP may alleviate PLSP by modulating core targets (e.g., COX-2, TNF-α) and signaling pathways (e.g., PI3K-Akt). These findings provide a systemic mechanistic hypothesis and a focused direction for subsequent experimental studies to confirm the bioactivity and therapeutic relevance of these predictions.

## 1. Introduction

Post-laparoscopic shoulder pain (PLSP) is a common postoperative complication, characterized by mild to moderate dull pain in the shoulder region that typically initiates within a few hours after surgery and can persist for 2 to 3 days.^[1]^ Beyond patient discomfort, PLSP can significantly impede postoperative recovery by limiting early mobilization, increasing the reliance on opioid analgesics with their associated side effects, and potentially prolonging hospital stays.^[2,3]^ The pathogenesis of PLSP is multifactorial, primarily attributed to referred pain from diaphragmatic irritation caused by residual carbon dioxide and surgical trauma, which involves complex inflammatory and neurogenic pathways.^[4]^ Effective management of PLSP remains a clinical challenge, highlighting the need for exploring complementary therapeutic strategies.

In traditional Chinese medicine (TCM), PLSP following laparoscopic surgery is often attributed to the invasion of “Cold” and “Dampness” pathogens, coupled with surgical trauma that leads to “Qi stagnation and Blood stasis” in the meridians (collaterals).^[5]^ This disrupts the normal flow of Qi and Blood, resulting in “obstruction causing pain.” The herbal formula investigated in this study is the hospital-prepared Wenjing Tongluo Powder (WTP; Warm Meridians and Unblock Collaterals Powder). It is composed of mugwort leaves (Ai Ye), Cinnamomi Ramulus (Gui Zhi), Lysimachia root (Jin Qian Cao), *Angelica pubescens* (Du Huo), sea balm bark (Hai Tong Pi), *Angelica dahurica* (Bai Zhi), *Siberian ginseng* root bark (Wu Jia Pi), perilla leaves (Zi Su Ye), *Sichuan pepper* (Hua Jiao), and *Citrus aurantium* (Zhi Ke). According to TCM principles, this formula is designed to warm the meridians, dispel cold and dampness, promote blood circulation, and relieve pain, thereby addressing the proposed TCM etiology of PLSP.^[6]^

However, the multicomponent, multi-target nature of TCM formulas poses a significant challenge for elucidating their modern pharmacological mechanisms using conventional reductionist approaches. Network pharmacology, grounded in systems biology theory, provides a powerful framework for this purpose. It systematically reveals interactions between drugs, their targets, and signaling pathways through molecular network construction and analysis, thereby helping to elucidate the relationship between active components and corresponding targets.^[7]^ Therefore, this study employs network pharmacology as a crucial first step to generate integrated, testable hypotheses. We aim to explore the potential therapeutic mechanisms of WTP by predicting its active compounds, key targets, and associated pathways in the context of PLSP. The systematic computational analysis conducted here is designed to prioritize the most promising mechanistic leads. These predictions will establish a focused roadmap and provide a solid theoretical foundation for subsequent in vitro and in vivo validation studies, which are planned as the next phase of this research program to confirm the biological and therapeutic relevance of the identified targets and pathways.

## 2. Method

### 2.1. Screening of active ingredients in Wenjing Tongluo Powder

This study utilized the Chinese Herbal Systematic Pharmacology Database Analysis Platform (https://www.tcmsp-e.com, accessed on January 11, 2025) to identify all chemical components from mugwort leaves, cinnamon twig, *Cynanchum stauntonii*, *A pubescens*, sea balm bark, *A dahurica*, *S ginseng* root bark, perilla leaves, *S pepper*, and *C aurantium*. The screening criteria were oral bioavailability ≥ 30% and drug-likeness ≥ 0.18 to identify key compounds with high pharmacological activity in the herbal compound formula, while excluding compounds without relevant information.

### 2.2. Target prediction of active components in Wenjing Tongluo Powder

Using the online database PubChem^[2]^ platform (https://pubchem.ncbi.nlm.nih.gov/, accessed on January 12, 2025) to obtain the structure-data file molecular structure information and simplified molecular input line entry system structural formulas of the compounds screened in the first step, then import the simplified molecular input line entry system format into the SwissTarget Prediction^[4]^ (http://www.swisstargetprediction.ch/, accessed on January 12, 2025) database, set the attribute to “Homo sapiens” and filter with the condition “probability > 0.”The target names were standardized by using the UniProt database^[5]^ (https://sparql.uniprot.org, accessed on January 12, 2025).

### 2.3. Acquisition of pain targets

Using the Online Mendelian Inheritance in Man database (https://omim.org, accessed on January 12, 2025),^[5]^ we searched for pain-related target genes using “Pain” as the keyword. We also supplemented the disease targets related to pain using the therapeutic target database (https://db.idrblab.net/ttd/, accessed on January 12, 2025),^[6]^ PharmGKB database (https://www.pharmgkb.org, accessed on January 12, 2025),^[8]^ and DrugBank database (https://go.drugbank.com, accessed on January 12, 2025).^[9]^ After removing duplicate data, we identified potential targets for pain.

### 2.4. *Build a protein–protein interaction network*

The targets of WTP and pain obtained by the above steps were analyzed Venny2..1.0 (http://www.liuxiaoyuyuan.cn, accessed on January 13, 2025) to obtain the common targets of WTP and pain, and the Venny diagram was drawn. Import common targets into the STRING database (https://string-db.org/, accessed on January 13, 2025),^[10]^ construct a protein interaction network, set the species to “Homo sapiens,” confidence level to 0.900, and configure parameters for “Free nodes in hidden networks.”

Then, the TSV format file downloaded from STRING database was imported into Cytoscape 3.10.2,^[11]^ and the core targets were screened using Cytohubba plug-in.^[12]^

### 2.5. Identification of core targets

The CytoHubba plug-in in Cytoscape3.10.2 (https://cytoscape.org/, accessed on January 16, 2025) evaluates each node gene using ten algorithms including Maximum Clique Centrality, Neighborhood Component Centrality, and Degree Centrality. The top 10 genes from each algorithm were compiled, and the frequency of each gene’s appearance across all 10 lists was counted. The 10 genes with the highest occurrence frequency were ultimately selected as the key hub genes.

### 2.6. GO and KEGG enrichment analysis

Using RStudio software (Boston), we converted the Symbol identifiers of intersecting genes obtained in step 4 into Entrez IDs to prepare for GO analysis and KEGEG analysis. Functional enrichment entries were screened using the criterion “*P*_adjust ≤ .05,” where a closer *P*-adjustment value indicates more reliable enrichment results. After screening, we extracted the top 10 entries ranked by Gene Ratio from 3 categories: biological processes, cellular components, and molecular functions. Bubble plots were generated using the dotplot function and bar charts created with the barplot function. The statistically significant results were saved as txt files. Pathway enrichment analysis was performed using the enrichKEGG function based on the Kyoto Encyclopedia of Genes and Genome (KEGG) database, with the screening threshold set at “*P*_adjust < .01” to ensure more reliable and significant enrichment results. Visualization of the enrichment results was conducted using the ggplot2R package in R software.

### 2.7. Strategy for future experimental validation

Rationale and overall design: to experimentally test the core predictions generated by the network pharmacology analysis above, a 2-phase validation strategy is proposed, encompassing in vitro and in vivo studies.

#### 2.7.1. In vitro validation (anti-inflammatory activity)

##### 2.7.1.1. Cell model

Lipopolysaccharide (LPS)-stimulated RAW 264.7 murine macrophages.

##### 2.7.1.2. Experimental groups

Control group; LPS model group; LPS + WTP medicated serum (low/medium/high dose) groups; LPS + positive control (e.g., Dexamethasone) group.

##### 2.7.1.3. Outcome measures

Cell viability (CCK-8 assay); secretion levels of predicted core inflammatory targets such as tumor necrosis factor-alpha (TNF-α), interleukin-6, and prostaglandin-endoperoxide synthase 2 (PTGS2; cyclooxygenase-2 [COX-2]; measured by ELISA and/or Western Blot [WB]).

##### 2.7.1.4. Statistical analysis

Data will be expressed as mean ± standard deviation. Comparisons among multiple groups will be performed by 1-way analysis of variance followed by Tukey’s post hoc test. A *P*-value < .05 will be considered statistically significant.

#### 2.7.2. In vivo validation (analgesic efficacy and mechanism)

##### 2.7.2.1. Animal model

A established rat model of PLSP induced by diaphragmatic irritation with capsaicin or acidic saline.

##### 2.7.2.2. Experimental groups

Sham-operated group; PLSP model group; PLSP + WTP administration (low/medium/high dose) groups; PLSP + positive drug (e.g., Indomethacin) group.

#### 2.7.3. Outcome measures

##### 2.7.3.1. Behavioral pain score

Mechanical withdrawal threshold (von Frey filaments) and thermal hyperalgesia (hot plate test) at the shoulder region.

##### 2.7.3.2. Molecular and histological analysis

Protein expression levels of predicted hub targets (e.g., p-AKT/AKT, CASP3) in spinal cord dorsal horn or diaphragmatic tissue (WB). Histopathological examination of the affected diaphragm (hematoxylin and eosin staining).

##### 2.7.3.3. Statistical analysis

Similar to in vitro analysis. Behavioral data over time will be analyzed using 2-way repeated measures analysis of variance.

### 2.8. Ethical statement

This study is a computational network pharmacology analysis based entirely on public database mining and in silico predictions. No human participants, animal subjects, or personal clinical data were involved in the current research. Therefore, ethical approval from an institutional review board and informed consent were not required for this part of the work.

The proposed experimental validation strategies outlined in Section 2.7 represent planned future studies. For those future in vitro and in vivo experiments, all procedures will be designed and conducted in strict accordance with relevant ethical guidelines. Approval will be obtained from the Animal Care and Use Committee of Zhejiang University of Chinese Medicine (or the appropriate institutional ethics committee) prior to the commencement of any animal studies. All efforts will be made to minimize animal suffering.

## 3. Outcome

### 3.1. Screening of active components and exploration of corresponding targets of Wenjing Tongluo Powder

In the Traditional Chinese Medicine Systems Pharmacology Database and Analysis Platform database, screening criteria of ≥30% oral bioavailability and ≥0.18 drug-likeness were established to identify active components for warming meridians, unblocking collaterals, and dispersing pathogens. This process yielded 9 active components from mugwort leaves, 7 from cinnamon twigs, 5 from *C. stauntonii*, 9 from *A pubescens*, 8 from sea buckthorn cortex, 22 from *A dahurica*, 10 from Acanthopanax senticosus, 14 from perilla leaves, 5 from *S pepper*, and 5 from *C aurantium*. After removing duplicates (Table 1), SMILE structures were exported from PubChem and SwissTargetPrediction was performed to identify drug targets. Components with unpredictable targets were excluded, ultimately yielding 546 pharmacologically active targets.

**Table 1:** Seventy-two kinds of active ingredients of Wenjing Tongluo Powder screened from TCMSP database.

Molecule name	OB	OB	DL
MOL001040	(2R)-5,7-dihydroxy-2-(4-hydroxyphenyl)chroman-4-one	42.36	0.21
MOL001494	Mandenol	42	0.19
MOL002883	Ethyl oleate (NF)	32.4	0.19
MOL000358	beta-sitosterol	36.91	0.75
MOL000449	Stigmasterol	43.83	0.76
MOL005720	24-Methylenecyloartanone	41.11	0.79
MOL005735	Dammaradienyl acetate	44.83	0.83
MOL005741	Cycloartenol acetate	41.11	0.8
MOL000098	Quercetin	46.43	0.28
MOL001736	(−)-Taxifolin	60.51	0.27
MOL000359	Sitosterol	36.91	0.75
MOL000492	(+)-Catechin	54.83	0.24
MOL000073	ent-Epicatechin	48.96	0.24
MOL004576	Taxifolin	57.84	0.27
MOL011169	Peroxyergosterol	44.39	0.82
MOL000392	Formononetin	69.67	0.21
MOL005419	(6E,8E,10Z,12Z,14E,16E,18E,20Z,22Z,24E,26E)-2,6,10,14,19,23,27,31-octamethyldotriaconta-2,6,8,10,12,14,16,18,20,22,24,26,30-tridecaene	45.51	0.51
MOL005421	Alpha-Onocerin	39.31	0.73
MOL001941	Ammidin	34.55	0.22
MOL001942	Isoimperatorin	45.46	0.23
MOL003608	O-acetylcolumbianetin	60.04	0.26
MOL004777	Angelol D	34.85	0.34
MOL004778	[(1R,2R)-2,3-dihydroxy-1-(7-methoxy-2-oxochromen-6-yl)-3-methylbutyl] (Z)-2-methylbut-2-enoate	46.03	0.34
MOL004780	Angelicone	30.99	0.19
MOL004782	[(1R,2R)-2,3-dihydroxy-1-(7-methoxy-2-oxochromen-6-yl)-3-methylbutyl] 3-methylbutanoate	45.19	0.34
MOL004792	Nodakenin	57.12	0.69
MOL000443	Erythraline	49.18	0.55
MOL000448	Isobavachin	54.44	0.32
MOL000451	Erysodienone	37.29	0.44
MOL000453	1,2,6,7-Tetradehydro-3,15-dimethoxyerythrinan-16-ol	37.31	0.39
MOL000455	Erysotrine	113.82	0.44
MOL000456	Phaseolin	78.2	0.73
MOL000457	Phaseollidin	52.04	0.53
MOL001939	Alloisoimperatorin	34.8	0.22
MOL001956	Cnidilin	32.69	0.28
MOL005789	Neobyakangelico l	36.18	0.31
MOL005792	{5-[2′(R)-Hydroxy-3′-methyl-3′-butenyl-oxy]furocoumarin}	42.85	0.26
MOL005800	Byakangelicol	41.42	0.36
MOL005802	Propyleneglycol monoleate	37.6	0.26
MOL005806	4-[(2S)-2,3-dihydroxy-3-methylbutoxy]furo[3,2-g]chromen-7-one	39.99	0.29
MOL005807	sen-byakangelicol	58	0.61
MOL000953	CLR	37.87	0.68
MOL001506	Supraene	33.55	0.42
MOL001749	ZINC03860434	43.59	0.35
MOL002644	Phellopterin	40.19	0.28
MOL003588	Prangenidin	36.31	0.22
MOL003791	Linolein, 2-mono-	37.28	0.3
MOL007514	Methyl icosa-11,14-dienoate	39.67	0.23
MOL013430	Prangenin	43.6	0.29
MOL013110	Kaurenoic acid	59.52	0.34
MOL001645	Linoleyl acetate	42.1	0.2
MOL002575	Butyl-2-ethylhexyl phthalate	44.52	0.22
MOL012254	Campesterol	37.58	0.71
MOL001987	β-sitosterol	36.91	0.75
MOL000519	Coniferin	31.11	0.32
MOL000422	Kaempferol	41.88	0.24
MOL001558	Sesamin	56.55	0.83
MOL000006	Luteolin	36.16	0.25
MOL005030	Gondoic acid	30.7	0.2
MOL006202	LAX	44.11	0.2
MOL002773	Beta-carotene	37.18	0.58
MOL006209	Cyanin	47.42	0.76
MOL006210	Eugenyl-β-d-glucopyranoside(cirtrusinc)	40.52	0.23
MOL001771	Poriferast-5-en-3beta-ol	36.91	0.75
MOL007179	Linolenic acid ethyl ester	46.1	0.2
MOL013271	Kokusaginin	66.68	0.2
MOL002663	Skimmianin	40.14	0.2
MOL002881	Diosmetin	31.14	0.27
MOL013381	Marmin	38.23	0.31
MOL002341	Hesperetin	70.31	0.27
MOL004328	Naringenin	59.29	0.21
MOL005828	Nobiletin	61.67	0.52

CLR = cholesterol, DL = drug-likeness, LAX = laxogenin, NF = National Formulary, OB = oral bioavailability, TCMSP = Traditional Chinese Medicine Systems Pharmacology Database and Analysis Platform.

### 3.2. Pain-related targets

With “pain” as the retrieval keyword, we collected 298 pain-related targets from DrugBank database, 131 from pharmgkb database, 16 from Online Mendelian Inheritance in Man database and 100 from therapeutic target database. After deleting duplicate genes, 471 pain targets were obtained.

### 3.3. Construction of the “Chinese medicine-disease target” network

**Figure 1.**
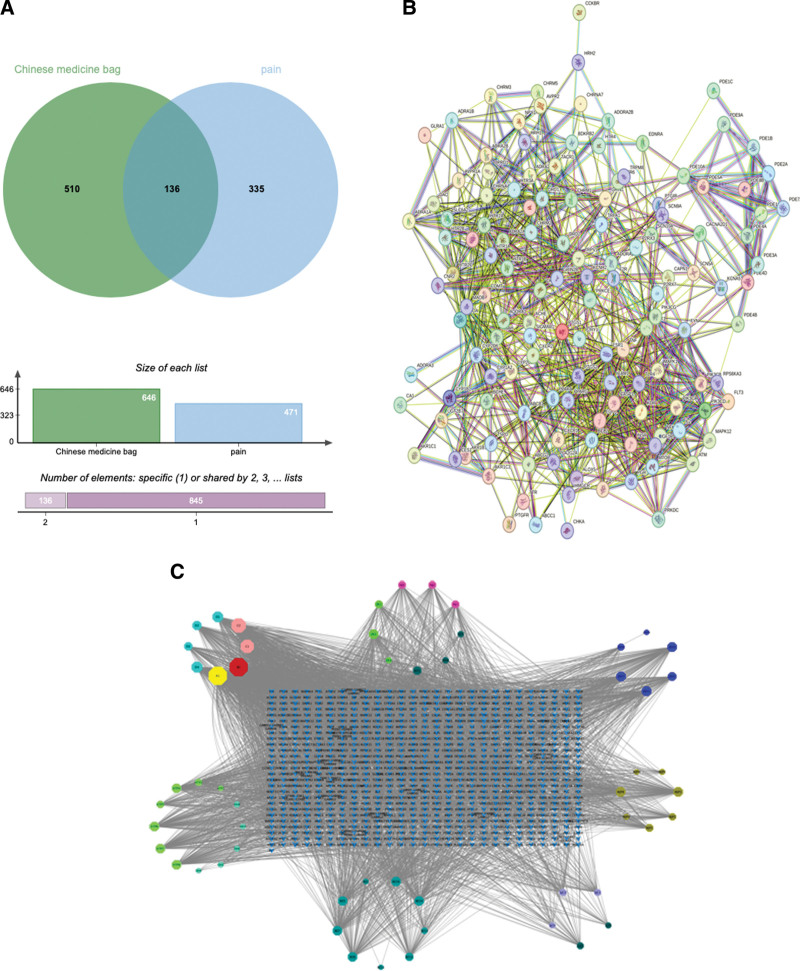
(A) Drug-disease common targets. (B) Drug-disease common target PPI network. (C) The “drug-component-disease-target” network relationship of Wenjingtongluo Powder for relieving PLSP in Figure C. (A) Drug-disease common targets. (B) PPI network of drug-disease common targets. (C) Wenjing Tongluo Powder alleviates PLSP with “drug - component - disease - target” network relationship. PLSP = post-laparoscopic shoulder pain, PPI = protein–protein interaction.

Using the Venny2.1.0 website to intersect the active components of WTP with pain targets, we identified 136 overlapping targets (Fig. 1A). These targets were then input into the STRING database with species set as “Homo sapiens” and confidence level set to 0.900, along with parameters for “hidden free nodes in networks” to obtain protein interaction data (Fig. 1B). By applying pinyin initials of TCM names and simplifying duplicate entries, we encoded each TCM formula and created “type” and “network” files mapping these 136 targets to their corresponding TCM names. The network was then imported into Cytoscape3.8.0 to construct a “TCM-disease-target network” (Fig. 1C), which contains 786 nodes and 3999 edges. The network diagram was configured using the “Column” option where node size corresponds to Degree values, with larger nodes indicating higher connectivity.

### 3.4. Selection and analysis of key targets

**Figure 2.**
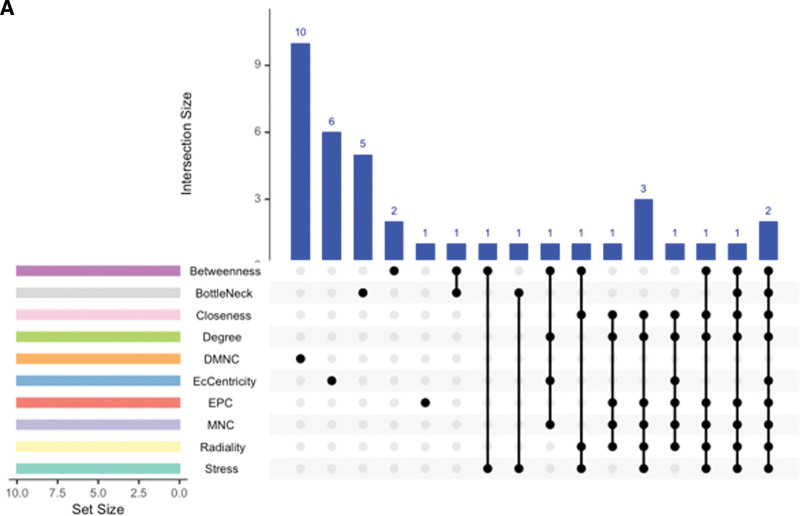
A Identification of core genes. The R package “Upset” ten algorithms for screening core genes. DMNC = density of maximum neighborhood component, EPC = edge percolated component, MNC = maximum neighborhood component.

Using Cytohubba’s 10 algorithms, we identified the top 10 hub genes (Fig. 2A). After intersection analysis using the Venny diagram, we identified 10 common key targets including COX-2, TNF, ESR1, EGFR, CASP3, BCL2, MAOA, MAOB, and PPARG.

### 3.5. GO and KEGG enrichment analysis

**Figure 3.**
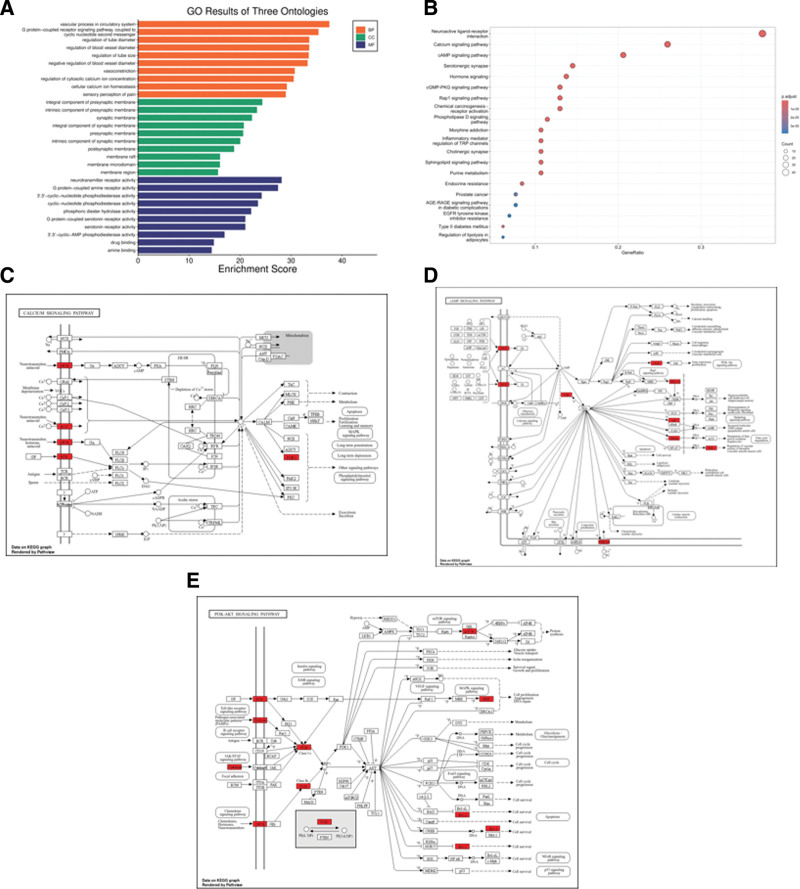
(A) A bar chart of the top 10 items of GO functional enrichment analysis for intersecting targets. Bubble diagram of the top 10 items of KEGG functional enrichment analysis of intersection targets in Figure B. (C–E) The pathways and key targets involved in the relief of PLSP by Wenjing Tongluo Powder. GO = Gene Ontology, KEGG = Kyoto Encyclopedia of Genes and Genomes, PLSP = post-laparoscopic shoulder pain.

To elucidate the potential biological processes and molecular functions of 136 intersected targets, we conducted GO and KEGG analyses using the R software package “clusterProfiler” with screening criteria of *P* < .05 and FDR < 0.05. Through GO enrichment analysis, a total of 1695 biological processes were identified (Fig. 3A). At the biological process level, WTP may reduce pain generation by regulating vascular diameter, muscle contraction, G protein-coupled receptors, and other processes. The KEGG enrichment analysis revealed 153 potential pathways (Fig. 3B). The top 10 enriched pathways (excluding cancer, parasitic, and infection-related pathways) suggest that WTP may alleviate pain through multiple mechanisms including calcium signaling pathways (Fig. 3C), cAMP signaling pathways (Fig. 3D), PI3K/AKT signaling pathways (Fig. 3E), and cGMP/PKG signaling pathways.

### 3.6. Integration of computational findings and implications for validation

The network pharmacology analysis above systematically predicted the potential active components, hub targets, and key signaling pathways involved in the action of WTP against PLSP. These computational results generate a series of testable hypotheses. Specifically, the predicted inhibition of core targets such as PTGS2 (COX-2) and TNF-α, and the modulation of central pathways like the PI3K-Akt and cAMP signaling pathways, constitute the primary mechanistic framework to be empirically validated in subsequent experimental studies. The following “Discussion” section will interpret these predictions in the context of existing biological knowledge and outline a strategic roadmap for their future verification.

## 4. Discussion

### 4.1. Summary of key findings and study rationale

PLSP is a prevalent complication that impedes postoperative recovery and reduces patient quality of life. While various physical maneuvers exist, their efficacy is often limited by patient compliance. WTP, a hospital-prepared TCM formulation, is used clinically to alleviate PLSP based on the TCM principle of “warming meridians and unblocking collaterals” to address pain caused by cold, dampness, and Qi/Blood stagnation. This study is the first to employ a network pharmacology approach to systematically predict the potential molecular mechanisms underlying its therapeutic effect. Our analysis identified 72 active components, 136 key targets (including COX-2, TNF, and CASP3), and several vital pathways (e.g., PI3K-Akt, cAMP) implicated in pain and inflammation. These computational results transform the formula’s traditional efficacy into a testable, multi-target mechanistic hypothesis, providing a prioritized roadmap for subsequent experimental validation.

### 4.2. Biological interpretation of predicted core targets and pathways

The pathogenesis of PLSP involves intricate interplay between inflammatory mediators and neuronal sensitization. Our predictions align coherently with established pain biology, lending credence to the hypotheses generated.

Hub targets as intervention points: the prediction of PTGS2 (COX-2) as a core target is highly plausible, as it is the rate-limiting enzyme in prostaglandin synthesis, a classic target for anti-inflammatory and analgesic drugs.^[12]^ TNF-α, another top-predicted target, is a master pro-inflammatory cytokine known to directly induce hyperalgesia and activate downstream pain-sensitizing pathways.^[13,14]^ Our finding that ingredients like *A dahurica* (Bai Zhi) may inhibit TNF-α release offers a plausible link between the formula’s composition and this predicted anti-inflammatory action.^[15]^ Furthermore, the inclusion of CASP3, a regulator of apoptosis, suggests the formula may also modulate cell death processes involved in inflammatory pain.^[16]^

Centrality of key signaling pathways: the significant enrichment of the PI3K-Akt signaling pathway is particularly noteworthy. This pathway is critically involved in both peripheral and central sensitization underlying chronic pain.^[17,18]^ Its prediction, alongside TNF-α (a known activator of PI3K/Akt^[14]^), indicates a potentially crucial node for the formula’s action. Similarly, the cAMP signaling pathway is a fundamental regulator of neuronal excitability and pain transduction.^[19]^ Our prediction suggests that WTP may modulate this pathway, analogous to the documented effects of other TCM formulas like Gui Zhi Tang,^[19]^ to attenuate pain signaling.

### 4.3. From computational predictions to a roadmap for experimental validation

To transform the above predictions into empirical evidence, we propose a concrete, 2-tiered experimental strategy.

#### 4.3.1. In vitro validation (anti-inflammatory efficacy)

The initial step will employ an LPS-stimulated RAW 264.7 macrophage model to test the predicted anti-inflammatory component of the mechanism. Experimental groups will include control, LPS model, and LPS plus WTP medicated serum (at varying concentrations). Key outcome measures will focus on the expression levels of predicted core targets, specifically TNF-α and COX-2 protein, using ELISA and WB analyses. This direct in vitro test will validate whether the formula’s components biologically engage the predicted primary inflammatory targets.

#### 4.3.2. In vivo validation (analgesic efficacy and pathway modulation)

To assess overall analgesic effect and pathway modulation, a validated rat model of PLSP (e.g., induced by diaphragmatic irritation) will be used. Groups will include sham, model, and formula treatment cohorts. Outcomes will combine behavioral pain assessments (e.g., mechanical withdrawal threshold at the shoulder) with molecular analysis of tissue from key sites (e.g., spinal cord dorsal horn). Crucially, WB analysis of the phosphorylation status of Akt (p-AKT/AKT ratio) will be performed to directly test the predicted modulation of the PI3K-Akt pathway in vivo.

### 4.4. Integration with traditional knowledge and added value of the study

The current predictions corroborate and systematize existing knowledge on individual herbs. For instance, the analgesic properties of cinnamaldehyde from Cinnamomi Ramulus (Gui Zhi)^[20]^ and *A dahurica* (Bai Zhi)^[21]^ are supported by pharmacological studies. However, the network pharmacology approach moves beyond a simple sum of parts. By revealing a “multicomponent, multi-target, multi-pathway” interactive network, it provides a systems-level hypothesis for the formula’s synergistic efficacy. This explains how the combined action of the entire formula, as suggested by studies on herb pairs like *S ginseng* bark and *Myrica rubra* bark,^[22]^ might yield superior therapeutic effects compared to single agents, offering a modern molecular perspective on TCM compatibility theory.

### 4.5. Limitations and future perspectives

This study has several limitations that define the scope of our current findings and guide future work:

#### 4.5.1. Inherent limitations of predictive methodology

The findings are contingent on the completeness and accuracy of the underlying databases. Furthermore, the ADME screening criteria, while standard, may have excluded some bioactive components.

#### 4.5.2. Need for empirical confirmation

The primary limitation is that all proposed mechanisms remain computational predictions. They must be considered hypothetical until rigorously tested through the proposed in vitro and in vivo validation studies outlined above. Future experimental work must also address potential limitations such as model specificity, sample size adequacy, and external validity.

#### 4.5.3. Path to clinical translation

Even successful experimental validation in models requires confirmation through randomized controlled clinical trials in patient populations to establish therapeutic efficacy definitively.

Future research should follow the translational pipeline from prediction to clinical application. After validating the core targets and pathways, techniques like molecular docking and gene knockdown could be used to delineate interactions and causal relationships in greater detail.

## 5. Conclusion

This study employed a systematic network pharmacology approach to predict the potential molecular mechanisms by which WTP alleviates PLSP. We identified a suite of active components, with key targets such as COX-2, TNF, and CASP3, and signaling pathways including the PI3K-Akt and cAMP pathways emerging as central to the predicted therapeutic effect. These findings provide a mechanistic hypothesis that bridges the formula’s traditional use of “warming meridians and unblocking collaterals” with modern biological understanding of pain and inflammation.

It is important to emphasize that the mechanisms proposed herein are derived from computational prediction. Therefore, the primary contribution of this work is to establish a prioritized and testable framework for future research. The integrated “multicomponent, multi-target, multi-pathway” perspective elucidated by this analysis offers a solid theoretical foundation and a clear experimental roadmap. Subsequent in vitro and in vivo studies, focused on validating the identified core targets and pathways, are essential to confirm these predictions and translate them into biological evidence. Ultimately, clinical-level evaluations will be indispensable to fully establish the therapeutic relevance and efficacy of WTP for PLSP management.

## Author contributions

**Conceptualization:** Hui Yu, Shen’ao Yu, Yanru Wang.

**Data curation:** Zhaoyang Wei.

**Formal analysis:** Zhaoyang Wei, Hui Yu, Yanru Wang.

**Investigation:** Zhaoyang Wei, Hui Yu, Yanru Wang.

**Methodology:** Zhaoyang Wei.

**Resources:** Zhaoyang Wei, Hui Yu, Yanru Wang.

**Software:** Zhaoyang Wei, Shen’ao Yu, Yanru Wang.

**Supervision:** Shen’ao Yu, Yanru Wang.

**Validation:** Yanru Wang.

**Visualization:** Zhaoyang Wei, Yanru Wang.

**Writing – original draft:** Zhaoyang Wei, Yanru Wang.

**Writing – review & editing:** Zhaoyang Wei, Yanru Wang.
